# Method to determine the tracking angles of heliostats

**DOI:** 10.1016/j.mex.2021.101244

**Published:** 2021-01-23

**Authors:** Victor Grigoriev, Kypros Milidonis, Manuel Blanco, Marios Constantinou

**Affiliations:** Energy, Environment and Water Research Center, The Cyprus Institute, 20 Konstantinou Kavafi Street, 2121 Nicosia, Cyprus

**Keywords:** Heliostat, Sun tracking, Solar tracker, Two-axis tracking, Dual-axis tracker

## Abstract

The heliostats with two tracking axes are considered, and the method is presented to find the tracking angles for reflection of sun light to a given target. An important advantage of the method is that the tracking axes are not required to be orthogonal like in azimuth-elevation, tilt-roll or target-aligned heliostats. All of these configurations are covered in a unified way, and the presented solution is valid even for arbitrary orientation of tracking axes. The ability to have such a general solution is very valuable, because the orthogonality condition may not hold precisely for manufacturing reasons or due to degradation of heliostats. These deviations need to be corrected properly to achieve a high concentration of sun light. The offsets between tracking axes are also taken into account. However, the targeting problem for heliostats in this case becomes considerably different from the inverse kinematic problems for robotic arm manipulators. It is shown that the tracking angles can be found iteratively, and the convergence of results is very fast for a typical set of parameters used in solar thermal plants. To simplify the use of the method, a Python-library HelioK was developed, and it is demonstrated how to work with it in a Jupyter-notebook. To explain the kinematics of heliostats better, a 3D model of heliostat is provided, which was made and animated in an open-source 3D editor Blender.

The main highlights of the method:

• The tracking axes and the facet of heliostat can have an arbitrary orientation, and there can be offsets between them.
• The tracking problem is solved both for targets attached to heliostat (local aiming) and for separated targets (global aiming).
• The single-axis trackers are included as a limiting case.

## Graphical abstract

**Figure:**
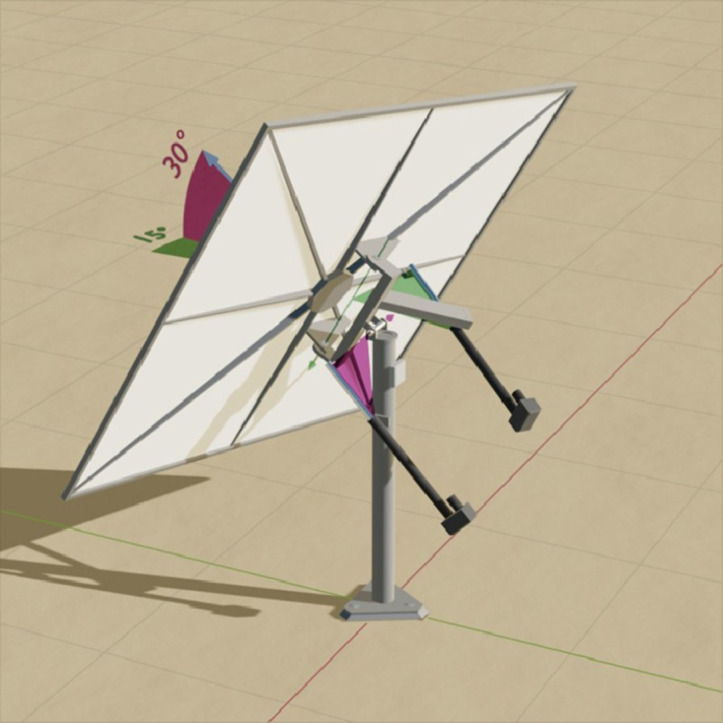

**Table:** Specifications Table

Subject Area	Energy
More specific subject area	*Concentrating Solar Power*
Method name	*Method to determine the tracking angles of heliostats*
Name and reference of original method	*Paden–Kahan subproblems for inverse kinematics*[2,3]
Resource availability	*HelioK repository:* https://scmt.cyi.ac.cy/bitbucket/projects/ART/repos/heliok *Anaconda (Python distribution):* https://www.anaconda.com/products/individual *Blender (3D editor):* https://www.blender.org/download

**Method details**

## Armature of heliostat

**Fig. 1:**
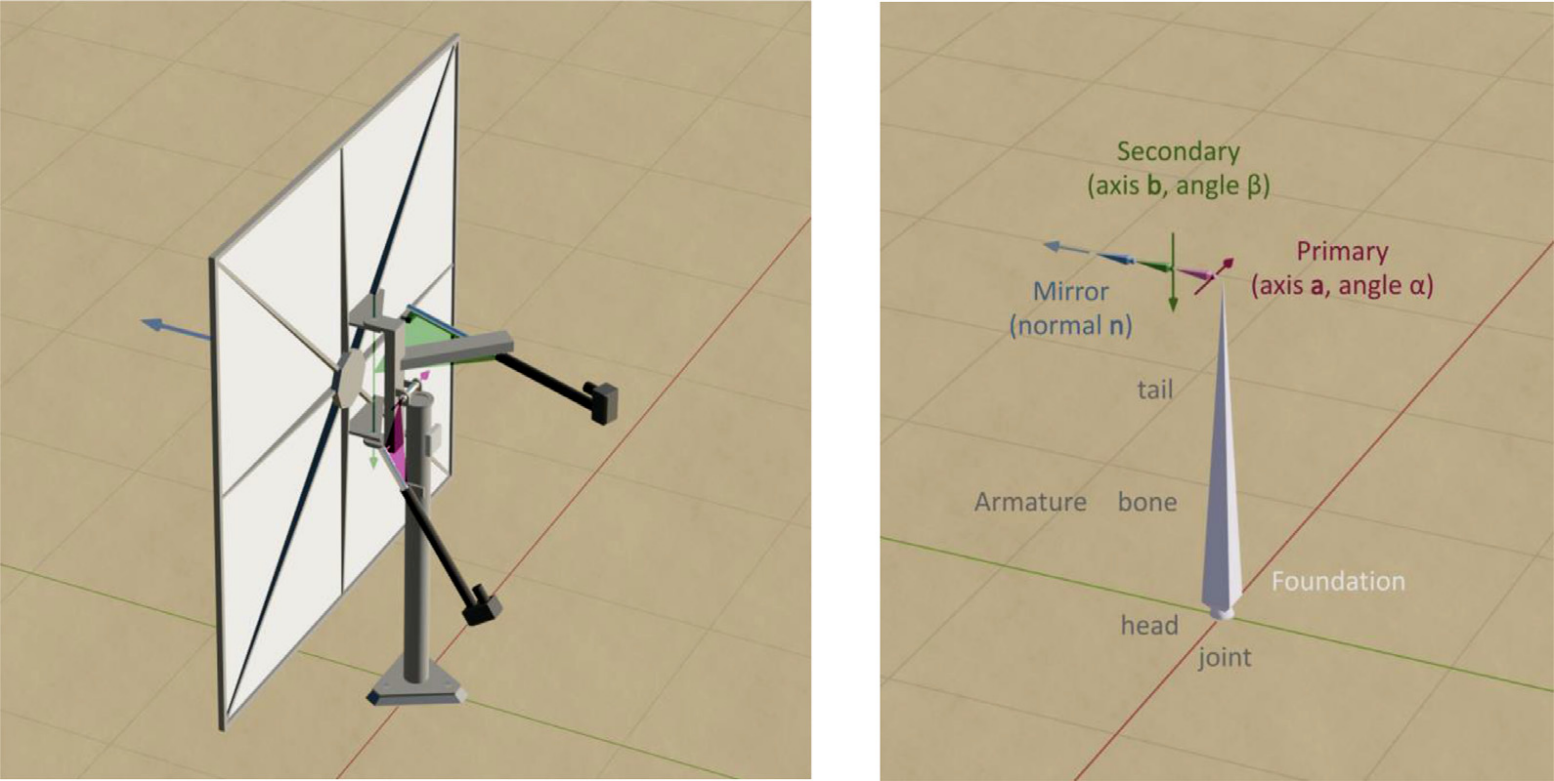
Heliostat with two tracking drives (a), and its armature (b). The arrows show the directions of primary (purple) and secondary (green) axes as well as the normal of mirror facet (blue). The rotation angles are controlled by changing the expansion length of linear actuators. The triangles show the rotation plane for each axis and the attachment points of linear actuators. The 3D model of heliostat is also provided as a supplementary material. (For interpretation of the references to colour in this figure legend, the reader is referred to the web version of this article.)

The rotation of heliostat is described by using an armature (Fig. 1). The armature consists of connected bones, which represent different parts of heliostat. The heads and tails of the bones can be linked by a hinge joint, which supports a rotation around given axis, or a more general joint type. The relative positions and orientations of the bones are defined in local heliostat frame when the primary and secondary angles are set to zero (Fig. 1a). The head of the foundation bone is positioned at the origin of heliostat frame. This bone is always fixed in the heliostat frame, and serves as a parent for other bones. The primary head has a translation $p_{a}$ from the foundation head. The hinge joint in the primary head can rotate around the axis a in the range $\left[\alpha_{\min},\;\alpha_{\max}\right]$. The secondary head has a translation $p_{b}$ from the primary head. The hinge joint in the secondary head can rotate around the axis b in the range $\left[\beta_{\min},\;\beta_{\max}\right]$. The rotation angles around the axes are measured according to the right-hand rule, and the static configuration (Fig. 1a) is used as a reference for zero angles. Notice that there is some ambiguity how to place the armature bones. Only the positions of heads are important where the hinges are, while the positions of tails can be selected to match the geometry of different moving parts. In particular, this was used to make the foundation bone vertical, although the primary axis is slightly shifted from it (Fig. 1b). The default directions of the primary and secondary bones can be used as a reference to define zero angles for the corresponding tracking axes. The mirror bone is fixed rigidly to the secondary bone, and it is necessary just to separate the mirror facet.

## Heliostat facets

In general, one or more mirror facets can be attached to the secondary bone. They can be described by appropriate translations $p_{m}$ and rotations $R_{m}$. It is necessary to define a vertex (a combination of point $r_{M0}$ and normal $n_{0}$), which can be used to determine the main direction of reflection. For a single facet, it usually corresponds to the center of the facet. The position $r_{M}$ and normal n of the tracking vertex depend on the primary α and secondary β angles as (the subscript ‘0’ is used when the values are taken for zero tracking angles)

(1) $$\left(\begin{array}{c} r_{M}\\ 1 \end{array}\right)=\left[\begin{array}{cc} R_{a}\left(\alpha \right) & p_{a}\\ 0 & 1 \end{array}\right]\left[\begin{array}{cc} R_{b}\left(\beta \right) & p_{b}\\ 0 & 1 \end{array}\right]\left(\begin{array}{c} r_{M0}\\ 1 \end{array}\right),$$

(2) $$n=R_{a}\left(\alpha \right)R_{b}\left(\beta \right)n_{0}.$$

## Heliostat drives

The positive rotation around the axes is determined by the right-hand rule. The rotation matrix around the axis a for angle α is computed as

(3) $$R_{a}\left(\alpha \right)=\cos\alpha \;I+\sin\alpha \;a^{\times }+\left(1-\cos\alpha \right)a⊗a,$$

where I is the identity operator, $a^{\times }$ is a cross product in matrix form, and $a⊗a=aa^{T}$ is a dyadic product. The rotation can be performed by slew drives or linear actuators. The former control the rotation angle directly, while the latter control the rotation angle via expansion length. If the distances from an axis to two attachment points are p and q, the expansion length λ according to the law of cosines is

(4) $$\lambda^{2}=p^{2}+q^{2}-2pq\cos\alpha .$$

This relation can be inverted to get the mapping α(λ).

## Heliostat field

The position and orientation of heliostat in global frame is defined by 4 × 4 transformation matrix

(5) $${\left(\begin{array}{c} r\\ 1 \end{array}\right)}_{\text{global}}=\left[\begin{array}{cc} R_{h} & p_{h}\\ 0 & 1 \end{array}\right]{\left(\begin{array}{c} r\\ 1 \end{array}\right)}_{\text{local}}.$$

The rotation matrix of heliostat can be decomposed into 3 Euler angles as $R_{h}=R_{x}\left(\varphi_{x}\right)R_{y}\left(\varphi_{y}\right)R_{z}\left(\varphi_{z}\right)$. In general, heliostats can be used to concentrate light on a target attached to it (local aiming) or a separated target (global aiming). A typical example of the former is a heliostat dish with a CPV (Concentrated Photovoltaics) element in the focus. The latter is mostly applied in CST (Concentrated Solar Thermal) power plants.

## Local aiming

The unit vector in the direction of the sun is converted to heliostat frame as $s\to R_{h}^{T}s$. Since the aiming point $r_{A}$ is specified in the same frame as the facet vertex $r_{M0}$, the target vector can be found as $t_{0}=\left(r_{A}-r_{M0}\right)/\left|r_{A}-r_{M0}\right|$, and it does not depend on the tracking angles. The corresponding sun vector according to the reflection law is $s_{0}=\left(2n_{0}⊗n_{0}-1\right)t_{0}$. The tracking angles are found as a rotation of $s_{0}$ to s (See Sequential rotation).

## Global aiming

The unit vector in the direction of the sun is converted to heliostat frame as $s\to R_{h}^{T}s$. The aiming point $r_{A}$ can be converted from global to heliostat frame as $r_{A}\to R_{h}^{T}\left(r_{A}-p_{h}\right)$. The target vector $t=\left(r_{A}-r_{M}\right)/\left|r_{A}-r_{M}\right|$, where for the first iteration $r_{M0}$ can be used instead of $r_{M}$. The required position of the normal according to the reflection law is n=(s+t)/|s+t|. The tracking angles are found as a rotation of $n_{0}$ to n (See Sequential rotation). Once the angles are found, they can be used to update $r_{M}$ according to Eq. (1). The accuracy of iteration is checked by substituting the new value of $r_{M}$ into $\left|t\times (r_{A}-r_{M})\right|$, which corresponds to the lateral displacement of reflected ray from target. Typically, a sub-millimeter accuracy is acceptable. Otherwise, a new iteration starts with updating the value of t.

## Sequential rotation

The primary α and secondary β angles for rotation of vector $v_{0}$ to v are found by composing an intermediate vector m, which separates the sequential rotation into two single-axis rotations [1]. The intermediate vector satisfies the equations a·v=a·m and $b\cdot m=b\cdot v_{0}$. It can be searched in the form $m=m_{a}a+m_{b}b\pm m_{k}k$ where k=a×b, and the solution of the system is

(6) $$\begin{array}{ccc} m_{a} & = & \frac{a\cdot v-\left(a\cdot b\right)\left(b\cdot v_{0}\right)}{1-{\left(a\cdot b\right)}^{2}},\\ m_{b} & = & \frac{b\cdot v_{0}-\left(a\cdot b\right)\left(a\cdot v\right)}{1-{\left(a\cdot b\right)}^{2}},\\ m_{k} & = & \frac{\text{sqrt}\left[1-m_{a}^{2}-m_{b}^{2}-2m_{a}m_{b}\left(a\cdot b\right)\right]}{\left|k\right|}. \end{array}$$

For each of the $m_{\pm }$ branches, the angles of single-axis rotations are found as

(7) $$\begin{array}{ccc} \alpha & = & \text{atan}\left[a\cdot m\times v,\;m\cdot v-{\left(a\cdot v\right)}^{2}\right],\\ \beta & = & \text{atan}\left[b\cdot v_{0}\times m,\;v_{0}\cdot m-{\left(b\cdot v_{0}\right)}^{2}\right]. \end{array}$$

By default, the angles are returned in the range [−π,π). It is possible to cast them to the range $\left[\varphi_{0},\varphi_{0}+2\pi \right)$ with the following transformation

(8) $$\varphi \to \varphi -2\pi \left\lfloor \frac{\varphi -\varphi_{0}}{2\pi }\right\rfloor ,$$

where ⌊⌋ is the floor function. This simplifies the check whether the angle is within a given range $\left[\varphi_{\min},\varphi_{\max}\right]$. It is also possible to introduce a function w(α,β)=|α|+|β| to sort the solutions and select the branch which requires less rotation.

## Single-axis trackers

The presented method can also be applied for single-axis trackers like troughs or Fresnel collectors (line focusing as opposed to point focusing). Due to the axial symmetry, it can be assumed that $n_{0}\cdot a=0$. The vectors s and t are projected on the rotation plane by using an operator 1−a⊗a and renormalizing the result. After that the similar steps (See Local aiming and Global aiming) can be applied to find the primary tracking angle α. The main difference is that rotation of vector $v_{0}$ to v is solved as

(9) $$\alpha =\text{atan}\left[a\cdot v_{0}\times v,\;v_{0}\cdot v\right].$$

## Implementation of method

### Input data

**Fig. 2:**
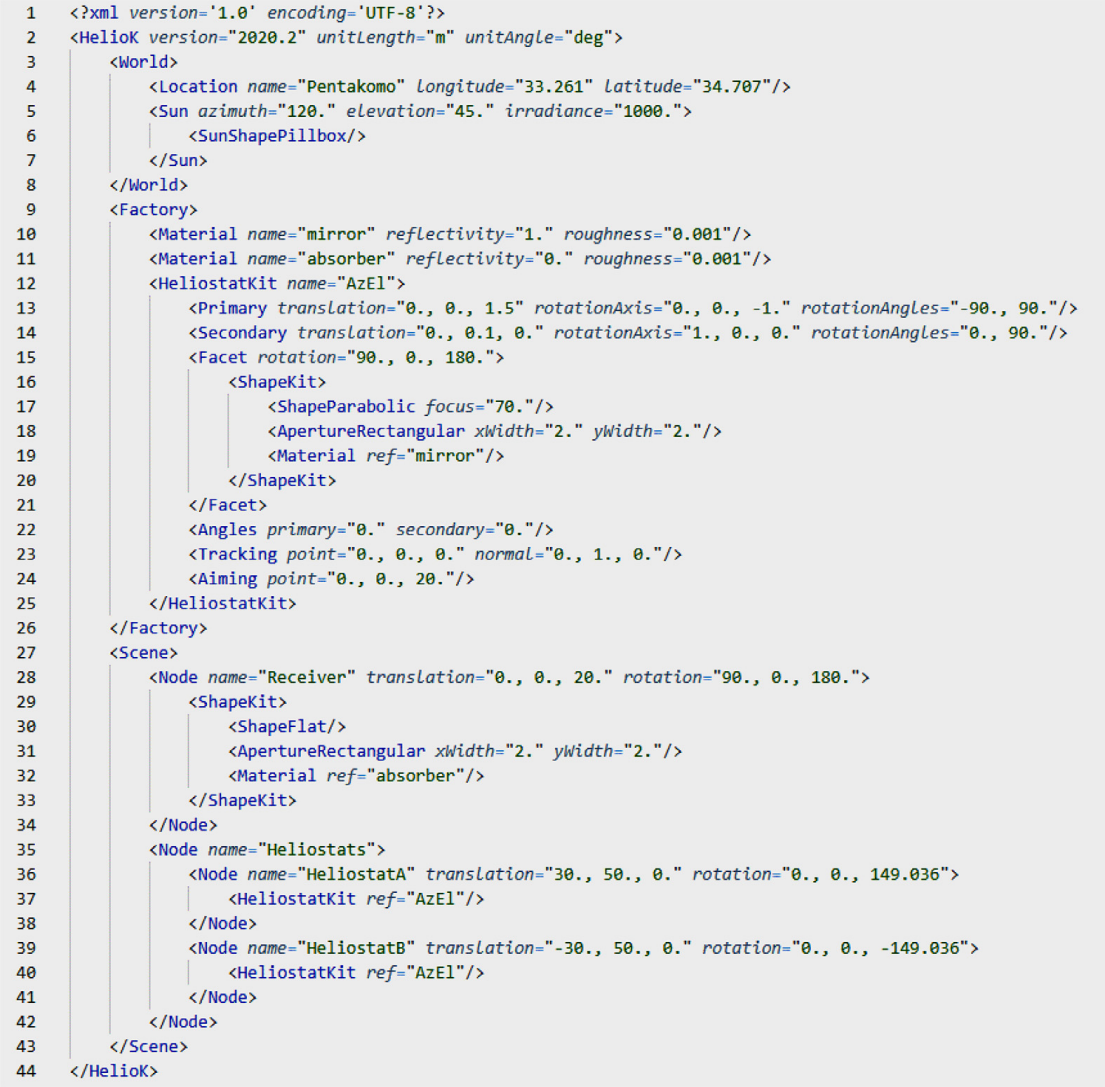
Definition of heliostat field in xml-format.

The method was implemented in Python and can be used as a library (HelioK). The parameters of heliostat field are stored in xml format (Fig. 2). The tag HelioK is used as a root and has the attributes to set the units of lengths and angles. The subtags World, Factory and provide the following details. The tag World defines the location of the plant and the position of the sun. The tag Factory stores the templates of materials and heliostats. The tag Scene describes the layout of the objects in the scene.

The parameters of heliostat are stored in the tag HeliostatKit and are grouped in the following way. The tag Primary with the attributes *translation* ($p_{a}$), *rotationAxis* (a) and *rotationAngles* ($\alpha_{\min},\;\alpha_{\max}$) is used for the primary drive. The tag Secondary with the attributes *translation* ($p_{b}$), *rotationAxis* (b) and *rotationAngles* ($\beta_{\min},\;\beta_{\max}$) is used for the secondary drive. The tag Facet with attributes *translation* ($p_{m}$, zero by default) and *rotation* ($R_{m}$, stored as Euler angles $\varphi_{x},\;\varphi_{y},\;\varphi_{z}$) defines a heliostat facet and can be repeated several times. The tag Angles with the attributes *primary* (α) and *secondary* (β) stores the orientation of heliostat. The tag Tracking with the attributes *point* ($r_{M0}$) and *normal* ($n_{0}$) defines a vertex on the facet which will be used for tracking. The tag Aiming with the attribute *point* ($r_{A}$) sets the aiming point.

The actual placement of heliostats is described in the tag Scene. It contains a tree of Node tags, which define geometrical transformations. Each node has the attributes *translation* (p) and *rotation* (R, stored as Euler angles $\varphi_{x},\;\varphi_{y},\;\varphi_{z}$). The nodes can be nested, which combines the transformations. The heliostats are attached to the nodes by using the tag HeliostatKit with the attribute *ref*, which refers to a specific template with the attribute *name*.

## Example of usage

**Fig. 3:**
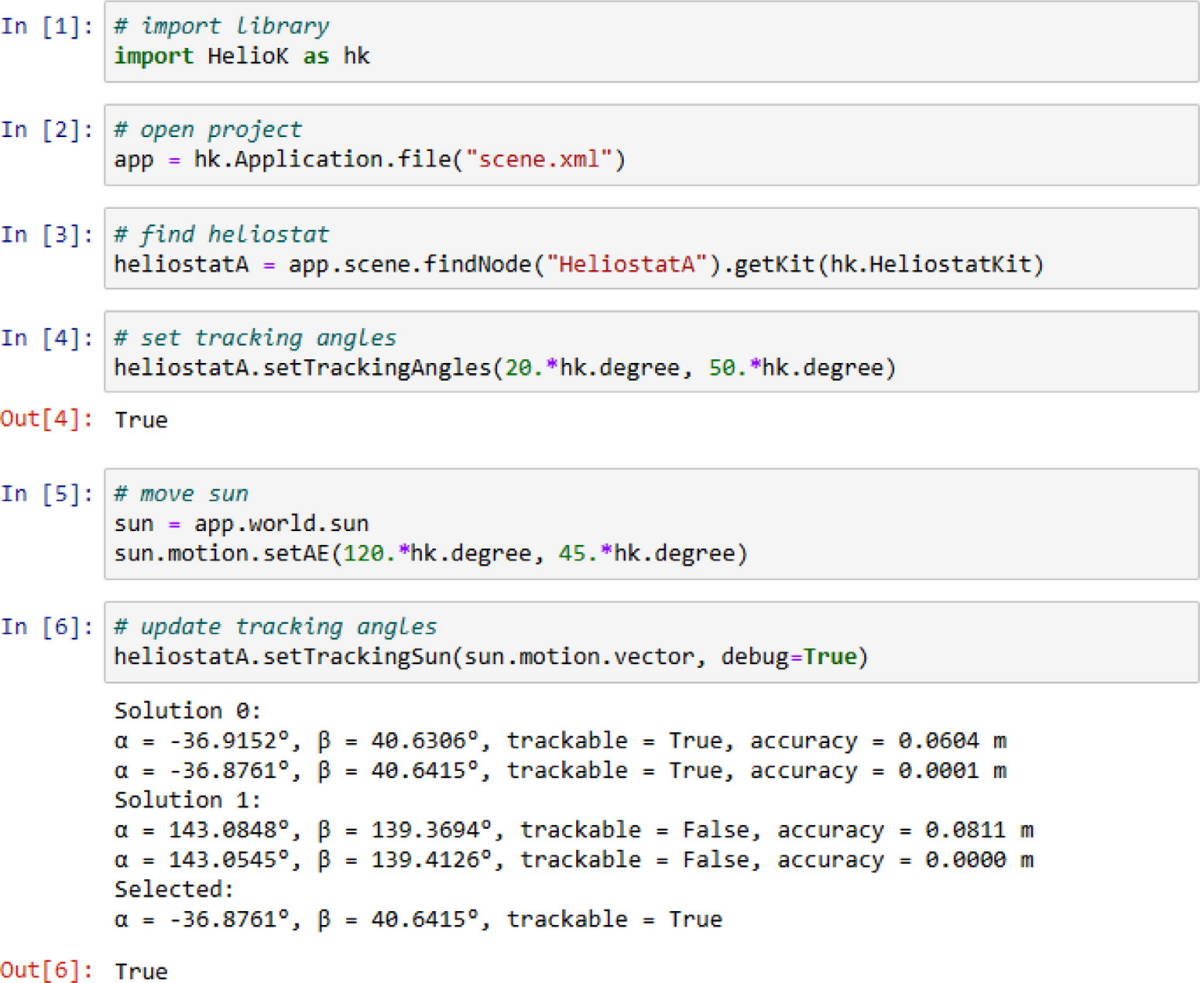
Example of using HelioK in Jupyter notebook.

The HelioK library can be used in Jupyter notebook (Fig. 3). The cell 1 imports the HelioK library with a short name hk. The cell 2 opens a heliostat field in xml-format and assigns it to a variable app. The cell 3 shows how to find a heliostat by name. The cell 4 sets the primary and secondary angles of the selected heliostat. The output is True, when the angles are within the tracking range. The cell 5 creates a reference to the sun object in the scene and changes its azimuth and elevation. The cell 6 updates the tracking angles of heliostat according to the position of the sun. The optional parameter debug enables a debug output. It shows two possible solutions and how the accuracy changes with iterations. If there is a solution within the tracking range, the tacking angles of heliostat are updated, and the output is True.

## Declaration of Competing Interest

The authors declare that they have no known competing financial interests or personal relationships that could have appeared to influence the work reported in this paper.

## Appendix Supplementary materials

Image, video 1

Image, application 2
